# Ethyl 2-amino-4-phenyl-4*H*-1-benzo­thieno[3,2-*b*]pyran-3-carboxyl­ate

**DOI:** 10.1107/S1600536811027152

**Published:** 2011-07-23

**Authors:** Adil Boughaleb, Hafid Zouihri, Said Gmouh, Abdelali Kerbal, Mohamed El yazidi

**Affiliations:** a Département de Chimie, Faculté des Sciences, Dhar Mehraz, BP 1796 Atlas, 30000 Fés, Morocco; b Laboratoire de Diffraction des Rayons X, Centre National pour la Recherche Scientifique et Technique, Rabat, Morocco; c Centre National pour la Recherche Scientifique et Technique, Rabat, Morocco

## Abstract

The title heterocyclic compound, C_20_H_17_NO_3_S, was synthesized by condensation of ethyl cyano­acetate with (*Z*)-2-benzyl­idenebenzo[*b*]thio­phen-3(2*H*)-one in the presence of a basic catalyst in ethanol. The phenyl and ester groups make dihedral angles of 77.67 (6) and 8.52 (6)°, respectively, with the benzothienopyran ring system [maximum r.m.s. deviation = 0.1177 (13) Å]. In the crystal, centrosymmetric dimers are formed through pairs of N—H⋯O hydrogen bonds between the amine and ester groups. Inter­molecular C—H⋯N hydrogen bonds and C—H⋯π inter­actions involving the thio­phene ring are also observed.

## Related literature

For general background to Michael addition reactions, see: Perlmutter (1992 ▶); Czarnocki *et al.* (2005 ▶); Rossiter & Swingle (1992 ▶). 
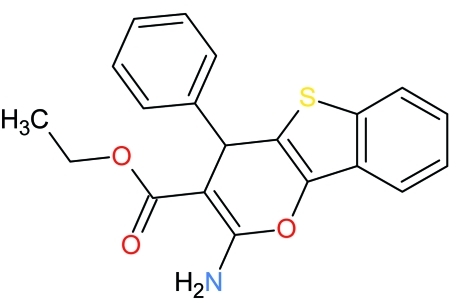


## Experimental

### 

#### Crystal data

C_20_H_17_NO_3_S
*M* *_r_* = 351.41
Monoclinic, 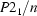

*a* = 8.6612 (3) Å
*b* = 5.9156 (2) Å
*c* = 32.3008 (10) Å
β = 94.962 (2)°
*V* = 1648.77 (9) Å^3^
*Z* = 4
Mo *K*α radiation
μ = 0.22 mm^−1^
*T* = 296 K
0.25 × 0.14 × 0.12 mm

#### Data collection

Bruker APEXII CCD detector diffractometer
21683 measured reflections
3608 independent reflections
3189 reflections with *I* > 2σ(*I*)
*R*_int_ = 0.031

#### Refinement

*R*[*F* ^2^ > 2σ(*F* ^2^)] = 0.034
*wR*(*F* ^2^) = 0.086
*S* = 1.04
3608 reflections
233 parameters
2 restraints
H atoms treated by a mixture of independent and constrained refinement
Δρ_max_ = 0.30 e Å^−3^
Δρ_min_ = −0.22 e Å^−3^

### 

Data collection: *APEX2* (Bruker, 2005 ▶); cell refinement: *SAINT* (Bruker, 2005 ▶); data reduction: *SAINT*; program(s) used to solve structure: *SHELXS97* (Sheldrick, 2008 ▶); program(s) used to refine structure: *SHELXL97* (Sheldrick, 2008 ▶); molecular graphics: *PLATON* (Spek, 2009 ▶); software used to prepare material for publication: *publCIF* (Westrip, 2010 ▶).

## Supplementary Material

Crystal structure: contains datablock(s) I, global. DOI: 10.1107/S1600536811027152/bh2365sup1.cif

Structure factors: contains datablock(s) I. DOI: 10.1107/S1600536811027152/bh2365Isup2.hkl

Supplementary material file. DOI: 10.1107/S1600536811027152/bh2365Isup3.cml

Additional supplementary materials:  crystallographic information; 3D view; checkCIF report

## Figures and Tables

**Table 1:** Hydrogen-bond geometry (Å, °) *Cg*1 is the centroid of the thio­phene ring.

*D*—H⋯*A*	*D*—H	H⋯*A*	*D*⋯*A*	*D*—H⋯*A*
N1—H1*B*⋯O2^i^	0.838 (19)	2.285 (19)	2.9143 (16)	132.1 (15)
C10—H10⋯N1^ii^	0.98	2.57	3.5189 (17)	164
C15—H15⋯*Cg*1^iii^	0.93	2.95	3.7493 (15)	145

Symmetry codes: (i) 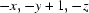; (ii) 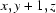; (iii) 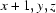.

**Fig. 1.**
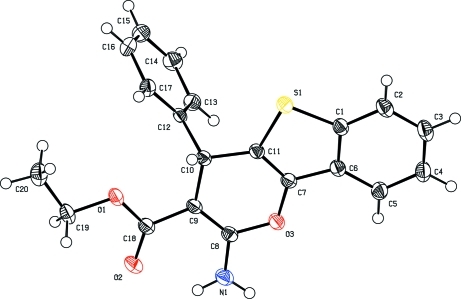
Molecular view of the title compound showing displacement ellipsoids at the 30% probability level. H atoms are represented as small spheres of arbitrary radii.

**Fig. 2.**
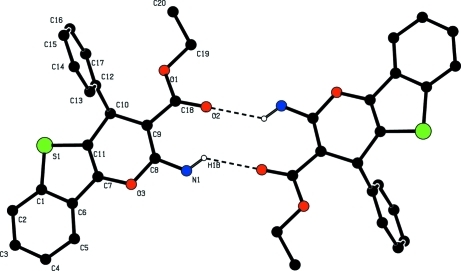
Partial view of the crystal packing, showing the N—H···O bonded dimers (symmetry code: -x, -y+1, -z). H atoms not involved in hydrogen bonds have been omitted for clarity.

## supplementary crystallographic information

### Comment

The Michael addition of carbanions to the C═C double bond of α,β-unsaturated ketones, nitriles, amides and esters is a method of choice for the formation of C—C bonds (Czarnocki *et al.*, 2005; Rossiter & Swingle, 1992; Perlmutter, 1992).

In this work we have studied the behavior of ethylcyanoacetate with respect to (*Z*)-2-benzylidenebenzo[*b*]thiophen-3(2*H*)-one and derivatives in ethanol, with the presence of piperidine as catalyst. We have shown that cyclocondensation starts with a Michael 1,4-addition, followed by intramolecular cyclization *via* nucleophilic addition of the hydroxyl group to the cyano group and not onto the carboxylate, to give the tricyclic heterocycle ethyl 2-amino-4-phenyl-4*H*-[1] benzothieno[3,2-*b*]pyran-3-carboxylate. The structural study by X-ray diffraction is in perfect agreement with the results of spectroscopic analysis: IR, ^1^H- and ^13^C-NMR.

In the title compound, C_20_H_17_NO_3_S (Fig. 1), the phenyl and ester groups make dihedral angles of 77.67 (6)° and 8.52 (6)°, respectively, with the benzothienopyran ring system. In the crystal, two molecules are linked about a center of inversion by pairs of N—H···O hydrogen bonds, generating a dimer (Fig. 2). Intermolecular C—H···N hydrogen bonds and C—H···π interactions (between C15—H15 bond of the phenyl group and the centroid of the thiophene ring with symmetry code: *x*+1, *y*, *z*) are also observed (Table 1).

### Experimental

In a 100 ml flask equipped with a condenser, was dissolved 4 mmol of (*Z*)-2-benzylidenebenzo[*b*]thiophen-3(2*H*)-one and 5 mmol of ethyl cyanoacetate in 30 ml of ethanol. Then, 1ml of piperidine was added, and the reaction mixture was refluxed for 6 h. Thin layer hromatography revealed the formation of a single product. The organic phase was evaporated under reduced pressure. The resulting residue was recrystallized from ethanol.

### Refinement

All H atoms bound to C atoms were treated as riding to their parent atoms [C—H distances are 0.93 Å for aromatic CH groups, 0.97 Å for methylene CH_2_ groups, 0.96 Å for the CH_3_ methyl group, and 0.98 Å for the CH methine group]. Isotropic displacement parameters are calculated as *U*_iso_(H) = 1.5 *U*_eq_(C20) for the methyl group and *U*_iso_(H) = 1.2 *U*_eq_(parent C) for other H atoms. The amine H atoms H1A and H1B were found in a difference map, and refined with N—H bond lengths restrained to 0.88 (2) Å and *U*_iso_(H) = 1.2 *U*_eq_(N1).

### Crystal data

**Table:**

C_20_H_17_NO_3_S	*F*(000) = 736
*M**_r_* = 351.41	*D*_x_ = 1.416 Mg m^−^^3^
Monoclinic, *P*2_1_/*n*	Mo *K*α radiation, λ = 0.71073 Å
Hall symbol: -P 2yn	Cell parameters from 232 reflections
*a* = 8.6612 (3) Å	θ = 2.6–25.3°
*b* = 5.9156 (2) Å	µ = 0.22 mm^−^^1^
*c* = 32.3008 (10) Å	*T* = 296 K
β = 94.962 (2)°	Prism, colourless
*V* = 1648.77 (9) Å^3^	0.25 × 0.14 × 0.12 mm
*Z* = 4	

### Data collection

**Table:**

Bruker APEXII CCD detector diffractometer	3189 reflections with *I* > 2σ(*I*)
Radiation source: fine-focus sealed tube	*R*_int_ = 0.031
graphite	θ_max_ = 27.0°, θ_min_ = 2.4°
ω and φ scans	*h* = −11→11
21683 measured reflections	*k* = −7→7
3608 independent reflections	*l* = −41→37

### Refinement

**Table:**

Refinement on *F*^2^	Primary atom site location: structure-invariant direct methods
Least-squares matrix: full	Secondary atom site location: difference Fourier map
*R*[*F*^2^ > 2σ(*F*^2^)] = 0.034	Hydrogen site location: inferred from neighbouring sites
*wR*(*F*^2^) = 0.086	H atoms treated by a mixture of independent and constrained refinement
*S* = 1.04	*w* = 1/[σ^2^(*F*_o_^2^) + (0.0386*P*)^2^ + 0.7884*P*] where *P* = (*F*_o_^2^ + 2*F*_c_^2^)/3
3608 reflections	(Δ/σ)_max_ = 0.001
233 parameters	Δρ_max_ = 0.30 e Å^−^^3^
2 restraints	Δρ_min_ = −0.22 e Å^−^^3^
0 constraints	

### Fractional atomic coordinates and isotropic or equivalent isotropic displacement parameters (Å^2^)

**Table:**

	*x*	*y*	*z*	*U*_iso_*/*U*_eq_	
S1	0.18188 (4)	1.17172 (6)	0.191397 (10)	0.02581 (10)	
O3	0.03012 (11)	0.62652 (16)	0.13342 (3)	0.0244 (2)	
C9	0.15146 (15)	0.8299 (2)	0.08005 (4)	0.0209 (3)	
C7	0.07313 (15)	0.8007 (2)	0.16060 (4)	0.0209 (3)	
C12	0.39539 (14)	1.0315 (2)	0.11368 (4)	0.0191 (3)	
C10	0.21849 (14)	1.0162 (2)	0.10886 (4)	0.0193 (3)	
H10	0.1785	1.1610	0.0978	0.023*	
C11	0.15652 (15)	0.9814 (2)	0.15042 (4)	0.0206 (3)	
C6	0.02270 (15)	0.8029 (2)	0.20170 (4)	0.0219 (3)	
C18	0.16810 (15)	0.8490 (2)	0.03571 (4)	0.0233 (3)	
C8	0.06481 (15)	0.6545 (2)	0.09321 (4)	0.0212 (3)	
C1	0.07339 (15)	1.0023 (2)	0.22215 (4)	0.0233 (3)	
C2	0.03563 (16)	1.0502 (3)	0.26251 (4)	0.0280 (3)	
H2	0.0692	1.1827	0.2759	0.034*	
C5	−0.06701 (16)	0.6470 (3)	0.22184 (4)	0.0263 (3)	
H5	−0.1011	0.5140	0.2087	0.032*	
C4	−0.10381 (16)	0.6953 (3)	0.26180 (4)	0.0300 (3)	
H4	−0.1633	0.5936	0.2756	0.036*	
C13	0.48520 (16)	0.8567 (2)	0.13167 (4)	0.0246 (3)	
H13	0.4379	0.7236	0.1392	0.030*	
C17	0.46835 (16)	1.2279 (2)	0.10227 (4)	0.0240 (3)	
H17	0.4097	1.3453	0.0899	0.029*	
C19	0.26683 (17)	1.0704 (3)	−0.01709 (4)	0.0287 (3)	
H19A	0.1676	1.0940	−0.0328	0.034*	
H19B	0.3159	0.9389	−0.0282	0.034*	
C3	−0.05272 (17)	0.8949 (3)	0.28169 (4)	0.0303 (3)	
H3	−0.0790	0.9234	0.3085	0.036*	
C16	0.62864 (17)	1.2511 (3)	0.10919 (5)	0.0294 (3)	
H16	0.6764	1.3835	0.1015	0.035*	
C14	0.64516 (16)	0.8792 (3)	0.13841 (4)	0.0292 (3)	
H14	0.7043	0.7608	0.1503	0.035*	
C15	0.71678 (16)	1.0774 (3)	0.12754 (4)	0.0305 (3)	
H15	0.8236	1.0935	0.1326	0.037*	
C20	0.36800 (19)	1.2749 (3)	−0.01999 (5)	0.0361 (4)	
H20A	0.3148	1.4059	−0.0110	0.054*	
H20B	0.3914	1.2954	−0.0483	0.054*	
H20C	0.4625	1.2542	−0.0026	0.054*	
O1	0.24544 (11)	1.03714 (17)	0.02657 (3)	0.0257 (2)	
O2	0.11868 (12)	0.71560 (19)	0.00872 (3)	0.0319 (2)	
N1	0.00146 (15)	0.4847 (2)	0.06999 (4)	0.0279 (3)	
H1A	−0.062 (2)	0.392 (3)	0.0809 (5)	0.034*	
H1B	0.009 (2)	0.484 (3)	0.0443 (6)	0.034*	

### Atomic displacement parameters (Å^2^)

**Table:**

	*U*^11^	*U*^22^	*U*^33^	*U*^12^	*U*^13^	*U*^23^
S1	0.02830 (19)	0.02802 (19)	0.02170 (17)	−0.00438 (14)	0.00562 (13)	−0.00647 (14)
O3	0.0311 (5)	0.0234 (5)	0.0187 (5)	−0.0041 (4)	0.0023 (4)	−0.0009 (4)
C9	0.0206 (6)	0.0245 (7)	0.0173 (6)	0.0016 (5)	−0.0005 (5)	−0.0016 (5)
C7	0.0192 (6)	0.0240 (7)	0.0191 (6)	0.0016 (5)	−0.0001 (5)	−0.0022 (5)
C12	0.0204 (6)	0.0240 (6)	0.0130 (5)	0.0008 (5)	0.0021 (5)	−0.0034 (5)
C10	0.0193 (6)	0.0209 (6)	0.0176 (6)	0.0024 (5)	0.0005 (5)	−0.0004 (5)
C11	0.0187 (6)	0.0243 (7)	0.0187 (6)	0.0019 (5)	0.0009 (5)	−0.0026 (5)
C6	0.0179 (6)	0.0284 (7)	0.0194 (6)	0.0054 (5)	0.0003 (5)	0.0005 (5)
C18	0.0218 (6)	0.0272 (7)	0.0206 (6)	0.0023 (5)	0.0000 (5)	−0.0018 (5)
C8	0.0211 (6)	0.0233 (7)	0.0185 (6)	0.0040 (5)	−0.0016 (5)	−0.0009 (5)
C1	0.0192 (6)	0.0297 (7)	0.0210 (6)	0.0042 (5)	0.0015 (5)	0.0004 (6)
C2	0.0274 (7)	0.0355 (8)	0.0209 (6)	0.0068 (6)	0.0016 (5)	−0.0035 (6)
C5	0.0233 (7)	0.0315 (7)	0.0241 (7)	0.0005 (6)	0.0011 (5)	0.0038 (6)
C4	0.0240 (7)	0.0422 (9)	0.0243 (7)	0.0034 (6)	0.0043 (5)	0.0094 (6)
C13	0.0254 (7)	0.0256 (7)	0.0227 (6)	0.0008 (5)	0.0007 (5)	0.0017 (6)
C17	0.0252 (7)	0.0249 (7)	0.0222 (6)	0.0017 (5)	0.0034 (5)	0.0021 (6)
C19	0.0335 (8)	0.0369 (8)	0.0161 (6)	0.0048 (6)	0.0034 (5)	0.0020 (6)
C3	0.0263 (7)	0.0460 (9)	0.0191 (6)	0.0104 (6)	0.0047 (5)	0.0023 (6)
C16	0.0266 (7)	0.0333 (8)	0.0292 (7)	−0.0055 (6)	0.0073 (6)	0.0016 (6)
C14	0.0246 (7)	0.0341 (8)	0.0282 (7)	0.0072 (6)	−0.0015 (6)	0.0021 (6)
C15	0.0192 (7)	0.0442 (9)	0.0283 (7)	−0.0003 (6)	0.0026 (5)	−0.0021 (7)
C20	0.0381 (9)	0.0423 (9)	0.0289 (8)	0.0019 (7)	0.0082 (6)	0.0084 (7)
O1	0.0322 (5)	0.0294 (5)	0.0157 (4)	−0.0024 (4)	0.0027 (4)	0.0003 (4)
O2	0.0390 (6)	0.0359 (6)	0.0206 (5)	−0.0061 (5)	0.0010 (4)	−0.0076 (4)
N1	0.0353 (7)	0.0262 (6)	0.0219 (6)	−0.0057 (5)	0.0001 (5)	−0.0029 (5)

### Geometric parameters (Å, °)

**Table:**

S1—C11	1.7371 (13)	C5—H5	0.9300
S1—C1	1.7422 (14)	C4—C3	1.398 (2)
O3—C8	1.3681 (16)	C4—H4	0.9300
O3—C7	1.3839 (16)	C13—C14	1.390 (2)
C9—C8	1.3694 (19)	C13—H13	0.9300
C9—C18	1.4561 (18)	C17—C16	1.394 (2)
C9—C10	1.5247 (18)	C17—H17	0.9300
C7—C11	1.3465 (19)	C19—O1	1.4516 (15)
C7—C6	1.4330 (18)	C19—C20	1.501 (2)
C12—C17	1.3879 (19)	C19—H19A	0.9700
C12—C13	1.3905 (19)	C19—H19B	0.9700
C12—C10	1.5293 (17)	C3—H3	0.9300
C10—C11	1.5022 (17)	C16—C15	1.383 (2)
C10—H10	0.9800	C16—H16	0.9300
C6—C5	1.402 (2)	C14—C15	1.386 (2)
C6—C1	1.404 (2)	C14—H14	0.9300
C18—O2	1.2253 (17)	C15—H15	0.9300
C18—O1	1.3448 (17)	C20—H20A	0.9600
C8—N1	1.3421 (18)	C20—H20B	0.9600
C1—C2	1.4000 (18)	C20—H20C	0.9600
C2—C3	1.377 (2)	N1—H1A	0.871 (19)
C2—H2	0.9300	N1—H1B	0.838 (18)
C5—C4	1.386 (2)		
			
C11—S1—C1	91.31 (7)	C5—C4—C3	120.89 (14)
C8—O3—C7	116.29 (10)	C5—C4—H4	119.6
C8—C9—C18	117.89 (12)	C3—C4—H4	119.6
C8—C9—C10	123.22 (11)	C14—C13—C12	120.55 (13)
C18—C9—C10	118.65 (12)	C14—C13—H13	119.7
C11—C7—O3	123.83 (12)	C12—C13—H13	119.7
C11—C7—C6	115.61 (12)	C12—C17—C16	120.65 (13)
O3—C7—C6	120.49 (12)	C12—C17—H17	119.7
C17—C12—C13	118.85 (12)	C16—C17—H17	119.7
C17—C12—C10	119.85 (12)	O1—C19—C20	107.10 (12)
C13—C12—C10	121.19 (12)	O1—C19—H19A	110.3
C11—C10—C9	107.45 (11)	C20—C19—H19A	110.3
C11—C10—C12	110.46 (10)	O1—C19—H19B	110.3
C9—C10—C12	115.47 (10)	C20—C19—H19B	110.3
C11—C10—H10	107.7	H19A—C19—H19B	108.5
C9—C10—H10	107.7	C2—C3—C4	121.53 (13)
C12—C10—H10	107.7	C2—C3—H3	119.2
C7—C11—C10	124.58 (12)	C4—C3—H3	119.2
C7—C11—S1	111.31 (10)	C15—C16—C17	120.08 (14)
C10—C11—S1	124.10 (10)	C15—C16—H16	120.0
C5—C6—C1	119.86 (12)	C17—C16—H16	120.0
C5—C6—C7	130.52 (13)	C15—C14—C13	120.20 (13)
C1—C6—C7	109.59 (12)	C15—C14—H14	119.9
O2—C18—O1	121.79 (12)	C13—C14—H14	119.9
O2—C18—C9	126.25 (13)	C16—C15—C14	119.65 (13)
O1—C18—C9	111.95 (11)	C16—C15—H15	120.2
N1—C8—O3	109.09 (12)	C14—C15—H15	120.2
N1—C8—C9	127.01 (13)	C19—C20—H20A	109.5
O3—C8—C9	123.90 (12)	C19—C20—H20B	109.5
C2—C1—C6	121.28 (13)	H20A—C20—H20B	109.5
C2—C1—S1	126.56 (12)	C19—C20—H20C	109.5
C6—C1—S1	112.16 (10)	H20A—C20—H20C	109.5
C3—C2—C1	117.90 (14)	H20B—C20—H20C	109.5
C3—C2—H2	121.1	C18—O1—C19	115.69 (11)
C1—C2—H2	121.1	C8—N1—H1A	119.0 (11)
C4—C5—C6	118.54 (14)	C8—N1—H1B	119.6 (13)
C4—C5—H5	120.7	H1A—N1—H1B	120.2 (17)
C6—C5—H5	120.7		

### Hydrogen-bond geometry (Å, °)

**Table:**

Cg1 is the centroid of the thiophene ring.

**Table:**

*D*—H···*A*	*D*—H	H···*A*	*D*···*A*	*D*—H···*A*
N1—H1B···O2^i^	0.838 (19)	2.285 (19)	2.9143 (16)	132.1 (15)
C10—H10···N1^ii^	0.98	2.57	3.5189 (17)	164.
C15—H15···Cg1^iii^	0.93	2.95	3.7493 (15)	145

Symmetry codes: (i) −*x*, −*y*+1, −*z*; (ii) *x*, *y*+1, *z*; (iii) *x*+1, *y*, *z*.
